# Targeted disruption of cubilin reveals essential developmental roles in the structure and function of endoderm and in somite formation

**DOI:** 10.1186/1471-213X-6-30

**Published:** 2006-06-20

**Authors:** Brian T Smith, Jason C Mussell, Paul A Fleming, Jeremy L Barth, Demetri D Spyropoulos, Marion A Cooley, Christopher J Drake, W Scott Argraves

**Affiliations:** 1 Department of Cell Biology and Anatomy Medical University of South Carolina, 173 Ashley Avenue, Charleston, SC 29425, USA; 2 Pathology and Laboratory Medicine Medical University of South Carolina, 173 Ashley Avenue, Charleston, SC 29425, USA

## Abstract

**Background:**

Cubilin is a peripheral membrane protein that interacts with the integral membrane proteins megalin and amnionless to mediate ligand endocytosis by absorptive epithelia such as the extraembryonic visceral endoderm (VE).

**Results:**

Here we report the effects of the genetic deletion of cubilin on mouse embryonic development. Cubilin gene deletion is homozygous embryonic lethal with death occurring between 7.5–13.5 days post coitum (dpc). Cubilin-deficient embryos display developmental retardation and do not advance morphologically beyond the gross appearance of wild-type 8–8.5 dpc embryos. While mesodermal structures such as the allantois and the heart are formed in cubilin mutants, other mesoderm-derived tissues are anomalous or absent. Yolk sac blood islands are formed in cubilin mutants but are unusually large, and the yolk sac blood vessels fail to undergo remodeling. Furthermore, somite formation does not occur in cubilin mutants. Morphological abnormalities of endoderm occur in cubilin mutants and include a stratified epithelium in place of the normally simple columnar VE epithelium and a stratified cuboidal epithelium in place of the normally simple squamous epithelium of the definitive endoderm. Cubilin-deficient VE is also functionally defective, unable to mediate uptake of maternally derived high-density lipoprotein (HDL).

**Conclusion:**

In summary, cubilin is required for embryonic development and is essential for the formation of somites, definitive endoderm and VE and for the absorptive function of VE including the process of maternal-embryo transport of HDL.

## Background

Cubilin is a 460-kDa peripheral membrane protein expressed by a number of absorptive epithelial cells including those of the renal proximal convoluted tubule, ileum and yolk sac extraembryonic visceral endoderm (VE) [1]. The first described function of cubilin was as the receptor for intrinsic factor-vitamin B_12_/cobalamin (Cbl), serving a critical role in the intestinal absorption of Cbl [2,3]. Cubilin was later shown to be an endocytic receptor for apolipoprotein A-I (apoA-I)/high density lipoprotein (HDL), mediating uptake of HDL in the kidney and VE [4,5]. Other cubilin ligands include albumin, transferrin, immunoglobulin light chains, vitamin D-binding protein, myoglobin, galectin-3 and Clara cell secretory protein [6].

Three cell surface integral membrane proteins have been shown to interact with cubilin. The first identified was megalin, an endocytic receptor belonging to the low density lipoprotein receptor (LDLR) family [7,8]. Megalin functions together with cubilin to mediate endocytosis of apoA-I/HDL, presumably facilitating endocytosis of the cubilin-apoA-I/HDL complex. The cation-independent mannose 6-phosphate/insulin-like growth factor II-receptor (CIMPR) is another endocytic receptor that binds to cubilin [9], although the functional significance of its interaction with cubilin remains to be established. The ~48-kDa type I transmembrane protein, amnionless (AMN), is the most recent integral membrane protein found to interact with cubilin [10]. AMN is essential for efficient transport of cubilin to the apical cell surface as well as for membrane anchoring of cubilin [11,12],

Given the fact that cubilin is expressed by trophectoderm and VE [13,14], it is believed to play an important role in maternal-embryonic transport of nutrients. Several additional pieces of evidence support this hypothesis. First, cubilin has been shown to mediate VE uptake of holoparticle HDL, HDL-associated cholesterol and apolipoprotein A-I [8,14]. Furthermore, the work of Sahali et al. [15] demonstrated that cubilin monoclonal antibodies infused into circulation of pregnant rats (9 dpc), bound to VE cubilin and induced a spectrum of developmental abnormalities and embryonic resorption within 24–48 hours of infusion. The abnormalities included retarded embryonic growth, craniofacial defects involving the eye, ear and neural tube, hydrocephaly and telencephalic hypoplasia. Similarly, growth retardation and morphological anomalies were obtained when rat embryos (10 dpc) were cultured *ex utero *in the presence of cubilin antibodies [16]. Here we characterize the consequences of targeted deletion of the mouse cubilin gene on embryonic development.

## Results

### Generation of cubilin^-/- ^mice

**Figure 1:**
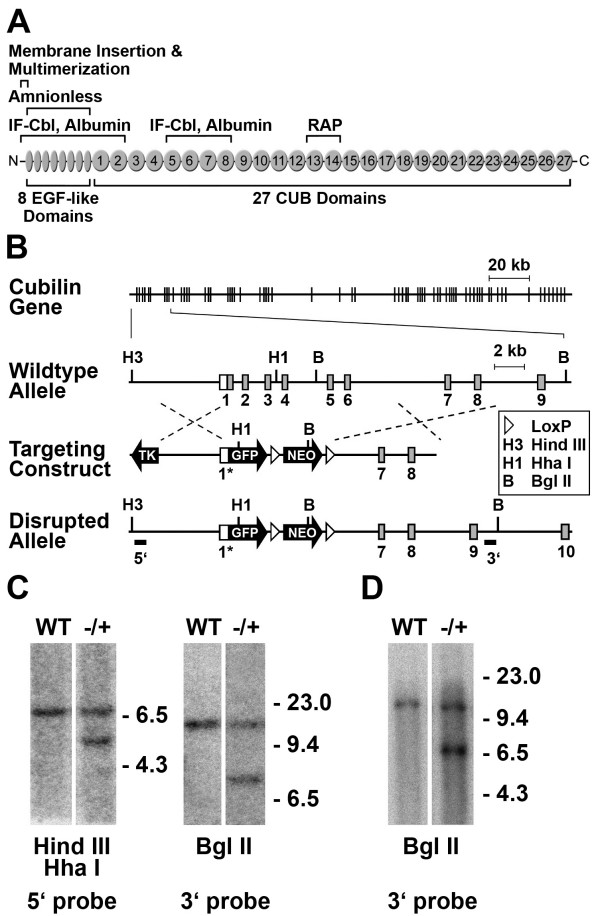
**Targeted deletion of the mouse cubilin gene**. ***A***, is a diagram of the structural and functional domains of cubilin. Ligand binding regions indicated in the diagram are based on published studies [12, 33, 34]. ***B***, is a diagram of the gene targeting strategy showing the organization of cubilin gene exons (vertical lines in uppermost model and rectangles in expanded region shown below), the wild-type and targeted alleles and the location of 5' and 3' DNA probes used for Southern blot analysis. White portions of boxed exons depict untranslated sequences. The promoter-less EGFP (containing Kozak and ATG sequences) and loxP-floxed neo^R ^cassettes were inserted into exon 1, 33 bp upstream of the cubilin ATG and 16 bp downstream from the proximal-most transcription initiation site, resulting in a replacement of the majority of exon 1. Homologous recombination between the targeting construct and the *cubilin *locus results in deletion of exon 1 coding sequence and all of exons 2–6. ***C***, Southern analysis of HindIII/HhaI and BglII digested DNA from representative wild-type (WT) and targeted (-/+) ES cell clones using 5' and 3' probes (left and right panels, respectively). The wild-type allele yields a 7 kb band and the knockout allele yields a 5.5 kb band when genomic DNA is digested with HindIII and HhaI and hybridized with the 5' probe. Additionally, the wild-type allele yields an 11.3 kb band and the knockout allele yields a 7 kb band when genomic DNA is digested with BglII and hybridized with the 3' probe. The results show that the correct recombinant allele is present in the selected ES cell clone. ***D***, Southern analysis of BglII digested tail DNA from a wild-type (WT) and a correctly targeted heterozygous (-/+) mouse.

To generate mice with targeted disruption of the cubilin gene (and concomitant knock-in of the EGFP reporter gene) we cloned and fully mapped the exon-intron structure of the 5' portion of the murine cubilin gene. A mouse cubilin gene-targeting vector was designed to create a null mutation through deletion of exons 1–6 (Fig. 1). After electroporation of the construct and G418 positive selection and FIAU negative selection, ten ES clones (out of 132 screened) were identified that had the desired recombination based on Southern analysis (using both upstream and downstream flanking probes) (Fig. 1). Targeted ES clones were injected into C57BL/6J blastocysts and the blastocysts were transferred to foster mothers to obtain chimeric mice. Two male chimeras were obtained from the first targeted ES cell line tested and found to be germ line competent through the generation of heterozygous offspring. As shown in Figure 1C, Southern analysis confirmed that offspring from one of these mice contained the properly targeted cubilin allele.

### The cubilin exon 1–6 deletion leads to embryonic lethality

Genotypic analysis was performed on 220 offspring from heterozygous intercross matings. As a result, 100 wild-type, 120 heterozygous and no homozygous offspring were detected (Table 1). These non-Mendelian ratios indicate that lethality occurs in embryos homozygous for the targeted cubilin gene deletion. Furthermore, since the frequency of heterozygotes at 4 weeks was much lower than expected (1:1 versus the expected 2:1 heterozygous:wild-type ratio), embryonic lethality appeared to be occurring in some heterozygotes. To substantiate this possibility we performed genotypic analysis of 181 4-wk offspring from wild-type × heterozygote matings. As a result, 109 wild-type and 72 heterozygous offspring were detected. Based on a chi-square test of these data and a resulting *p *value = 0.0074, the data are consistent with the hypothesis that haploinsufficiency of cubilin causes embryonic lethality, although with incomplete penetrance.

**Table I:** Genotype frequency of progeny from heterozygote (*cubilin *exon1–6^+/-^) matings

Stage	No. Progeny of Each Genotype			No. Resorbed Embryos	Total No.	Chi-Square *p *value
				
	+/+	+/-	-/-			
7.5 dpc	7	19	8	0	34	0.767
8.5 dpc	23	29	9	9	70	0.319
9.5 dpc	22	19	7	12	60	0.002
10.5 dpc	15	18	3	1	37	0.018
13.5 dpc	19	17	0	9	45	0.0001
Adult (4 wk)	100	120	0		220	0.0001*

Asterisk indicates that the chi-square test was based on a 1:2 ratio of wild-type to heterozygote offspring.

Retrograde genotypic analysis was performed on embryos from heterozygous intercross matings (Table 1). At 7.5 dpc homozygous embryos were obtained at a frequency consistent with a normal Mendelian ratio. From 8.5–10.5 dpc, homozygous embryos were detected, but not at a frequency in accordance with Mendelian expectations (Table 1). After 13.5 dpc, no homozygous embryos were detected. Together, these findings indicate that homozygous embryos die over a range of developmental stages between 7.5 to 13.5 dpc. Consistent with this conclusion was the relatively high number of embryo resorptions observed after 7.5 dpc (Table 1).

**Figure 2:**
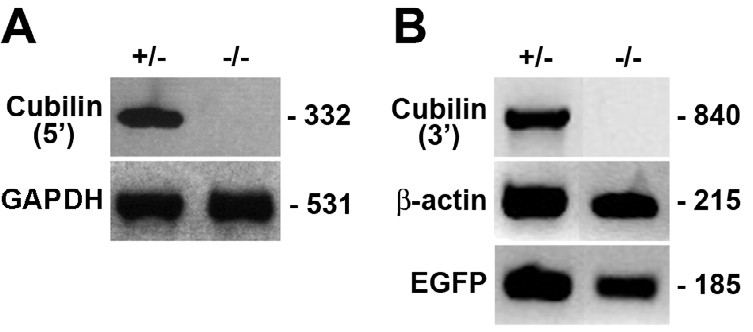
**Embryos homozygous for targeted deletion of *cubilin *do not express cubilin transcripts**. Shown are RT-PCR analyses of the expression of cubilin transcripts in RNA from heterozygous (+/-) and null (-/-) 8.5 dpc embryos. In ***A***, a primer pair corresponding to a 5' region of the cubilin transcript was used to assess cubilin transcript expression. In ***B***, a primer pair corresponding to a 3' region of the cubilin transcript was used to assess cubilin transcript expression.

To establish that embryos homozygous for the targeted cubilin gene deletion of exons 1–6 were indeed cubilin null, RT-PCR analysis was performed using two primer pairs designed to amplify separate 5' and 3' regions of the cubilin transcript. No cubilin mRNA was detectable by either primer pair using RNA isolated from homozygous 8.5 dpc embryos (Fig. 2). Furthermore, immunohistological analysis confirmed that there was no cubilin detectable in paraffin embedded sections of homozygous 8.5 dpc embryos (data not shown).

### Developmental retardation and mesodermal defects of homozygous embryos

**Figure 3:**
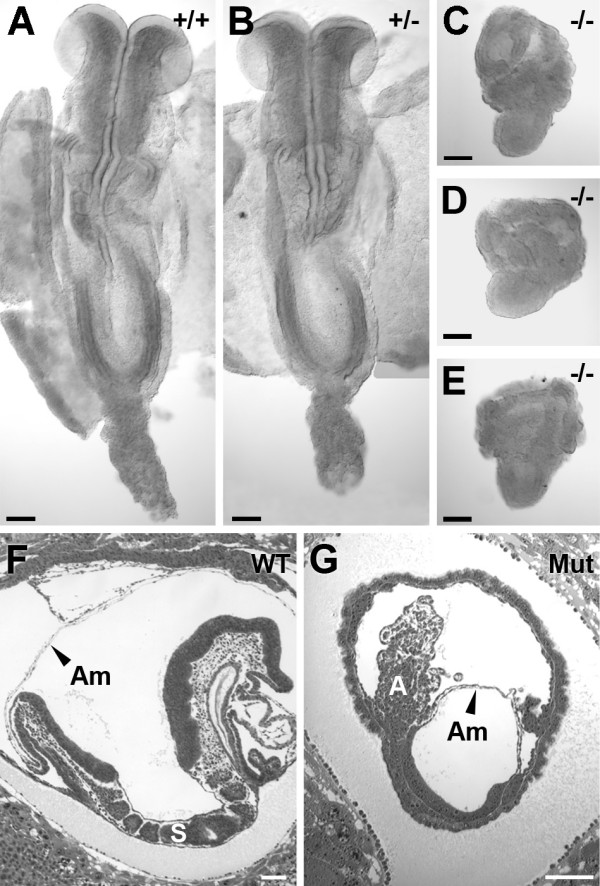
**Cubilin deficient 8–8.5 dpc embryos display growth retardation with formation of some mesodermal structures, but not somites**. Shown are wild-type (***A***), heterozygous (***B***) and homozygous 8–8.5 dpc embryos (C-E), derived from a heterozygous intercross mating. Embryos in A and B have been dissected to lie in a planar configuration where as embryos in C-D are intact. Shown in ***F ***and ***G ***are H&E stained sections of 8.5 dpc wild-type (F) and mutant (G) embryos. Mutant 8.5 dpc embryos possess an allantois and amnion, but lack somites. A, allantois; Am, amnion; S, somite. Bars in A-G = 100 μm.

Morphological analysis of 8–8.5 dpc embryos revealed that homozygous embryos were considerably smaller than wild-type or heterozygous littermates (Fig. 3). Mutant 8–8.5 dpc embryos were morphologically similar to wild-type 7.5 dpc embryos. However, rather than exhibiting a normal cylindrical shape, mutant 8–8.5 embryos appeared acorn shaped owing to a relatively smaller embryonic component as compared to extraembryonic component.

**Figure 4:**
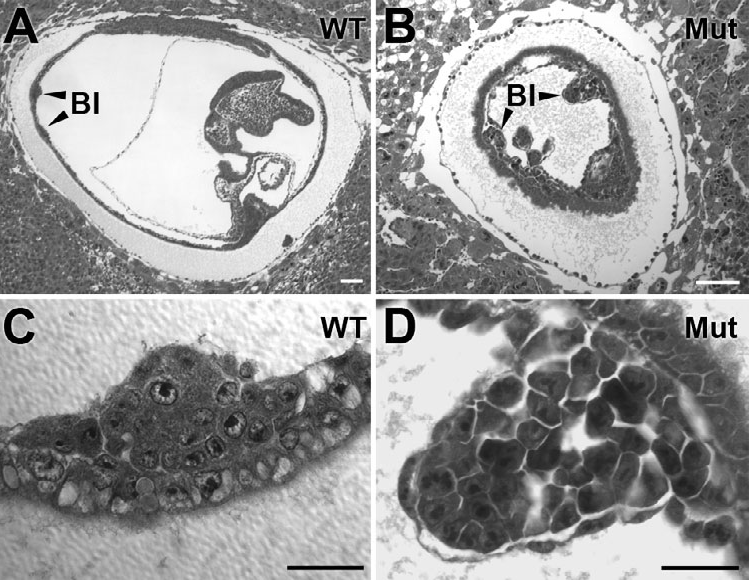
**Enlargement of blood islands in cubilin mutants**. Shown are H&E stained sections of an 8–8.5 dpc wild-type (***A***) and mutant (***B***) embryo. ***C ***and ***D ***show high magnification views of blood islands from embryos shown in *A *and *B*. Embryos in panels A and B are oriented such that anterior is to the right and posterior is to the left. BI, blood island. Bars in A and B = 100 μm. Bars in C and D = 25 μm.

Histological examination of null 8–8.5 dpc embryos revealed that all mutant embryos underwent gastrulation, but did not form somites (Fig. 3F and 3G). In contrast, 8–8.5 dpc wild-type littermates had formed 3–9 somites. Other mesodermal structures did form in the 8–8.5 dpc mutants, albeit with some variability. For example, an allantois was present in 5 of 7 mutant 8–8.5 dpc embryos examined. Most mutant embryos (5 of 7) also had an amnion and chorion, tissues having mesoderm components. While blood islands formed in the yolk sacs of all 8–8.5 dpc mutants they appeared unusually large, extending into the exocoelomic cavity to a greater extent than wild-type blood islands (Fig. 4). Such blood islands appeared to have a larger than normal number of mesodermal cells. Additionally, blood island hematopoietic development appeared to be retarded as evidenced by the failure of the cells to exhibit the characteristic rounded hematopoietic cell morphology (Fig. 4).

**Figure 5:**
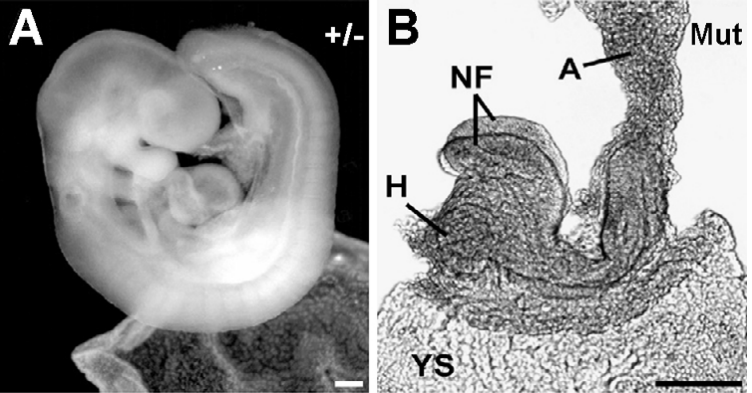
**Cubilin-deficient 9.5 dpc embryos are developmentally retarded and lack somites**. ***A ***shows a wild-type 9.5 dpc embryo and ***B ***a mutant littermate. NF, neural folds; A, allantois; H, heart; YS, yolk sac. Bars in A and B = 200 μm.

By 9.5 dpc, surviving homozygous embryos had not undergone axial rotation but displayed an overall morphological appearance of wild-type 8–8.5-dpc embryos (Fig. 5). Mutant 9.5 dpc embryos had formed a primitive heart and neural head folds (4 of 4 nulls examined) (Fig. 5) and paired dorsal aortae (data not shown), but lacked somites. Homozygous embryos surviving beyond 9.5 dpc (i.e., 11.5 dpc) were also grossly similar to wild-type 8.0 dpc embryos, but still lacked somites (data not shown). Together these findings indicate that lack of cubilin results in developmental retardation that initiates prior to 8.0 dpc, and a failure to achieve developmental milestones appropriate for a wild-type 8.0 dpc embryo, most notably, failure to form somites. The complete lack of somites at 9.5 dpc suggests that the basis is due to some defect of the paraxial mesoderm rather than merely being a consequence of retarded development.

It is important to note that embryos confirmed by genotyping to be heterozygous were indistinguishable morphologically from wild-type littermates over a range of stages examined extending from 7–11.5 dpc (Fig. 3B). This indicates that the lethality of heterozygous embryos, apparent from genotypic analysis, is occurring beyond 11.5 dpc.

### Failure of yolk sac blood vessels to undergo remodeling

**Figure 6:**
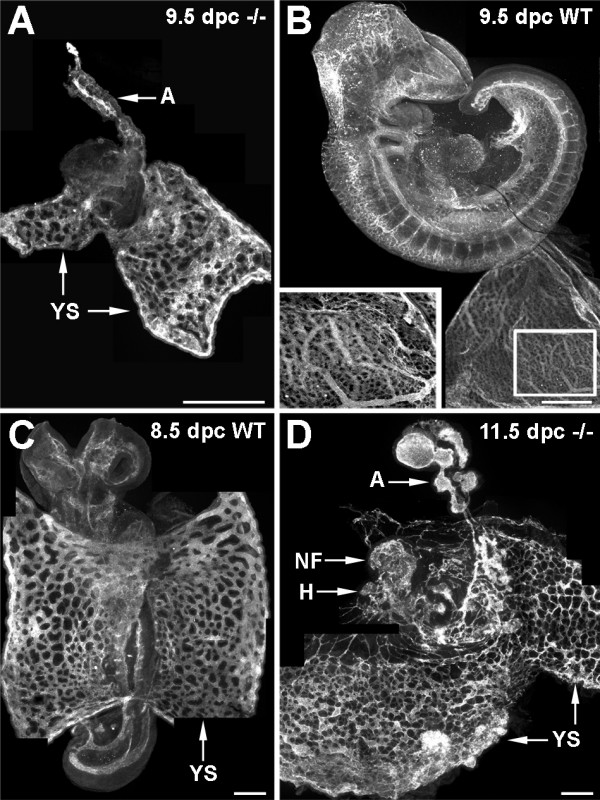
**Yolk sac blood vessels of 9.5 dpc cubilin mutants fail to undergo remodeling**. Anti-PECAM-1-labeled vasculature in a 9.5 dpc homozygous embryo (***A***), a 9.5 dpc wild-type (WT) embryo (littermate to that shown in *A*) (***B***), 8.5 dpc wild-type embryo (***C***) and 11.5 dpc homozygous embryo (***D***). The inset panel in *B *shows a higher magnification view of the remodeled yolk sac vasculature in the boxed region of the 9.5 dpc wild-type yolk sac. YS, yolk sac; A, allantois; NF, neural folds; H, heart. Bars in A and B = 500 μm. Bars in C and D = 200 μm.

To assess the effects on blood vessel formation, embryos from heterozygous matings were immunolabeled with anti-PECAM-1. As shown in Figure 6A, yolk sac blood vessel formation in 9.5 dpc mutants appeared retarded as compared to wild-type littermates (Fig. 6B). Normally by 9.5 dpc the yolk sac vessels of the wild-type embryo have undergone remodeling to form larger diameter vessels (Fig. 6B, *inset*). By contrast, the pattern of yolk sac vascular development in 9.5 dpc mutants appeared as an interconnected network of small diameter blood vessels (Fig. 6A), a pattern similar to that seen in an 8.5 dpc wild-type embryo yolk sac. In some areas of the extraembryonic yolk sac, PECAM-1 labeling often showed enlarged sinusoidal-like vessels (data not shown), which was consistent with the enlarged blood island-like structures observed in H&E stained sections (Fig. 4).

When the PECAM-1-labeled yolk sac vasculature of 11.5 dpc mutants was compared to that of wild-type embryos it was again evident that the blood vessels of homozygous embryos had not progressed beyond that of 8.5 dpc wild-type embryos. As shown in Figure 6D, yolk sac blood vessels of an 11.5 dpc mutant had not undergone the remodeling that normally occurs after 8.5 dpc.

In addition to the yolk sac vascular remodeling anomalies, the allantoic vasculature of 11.5 dpc mutants was aberrant. Instead of a branched central blood vessel normally present in an 8.5 dpc embryo [17], the allantoic blood vessels of 11.5 dpc mutants were bulbous and lacked branching (Fig. 6D). Since this abnormality was not observed in 9.5 dpc mutants, it can be concluded that dysmorphogenesis of the allantoic vasculature occurs after 9.5 dpc and perhaps contributes to a chorioallantoic placental defect.

### Abnormalities of embryonic and visceral endoderm in cubilin mutants

**Figure 7:**
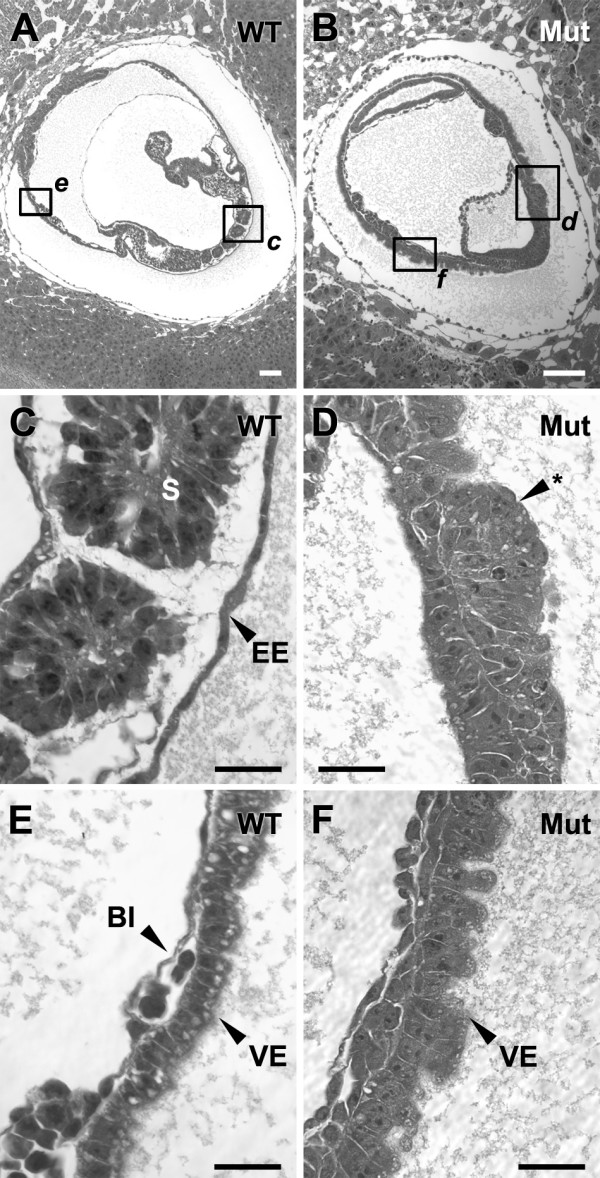
**Cubilin mutants display anomalies in the epithelial morphology of embryonic endoderm**. Shown are H&E stained sections of wild-type (***A, C ***and ***E***) and mutant (***B, D ***and ***F***) 8–8.5 dpc embryos. Embryos in panels A and B are oriented such that anterior is to the right and posterior is to the left. BI, blood island; S, somite; EE, definitive embryonic endoderm; VE, yolk sac visceral endoderm, Asterisk points to aberrant epithelium in place of the normal EE. Bars in A-F = 100 μm.

Considering that cubilin is normally present on the apical surface of the columnar VE (from 6.0 dpc to at least 9.5 dpc) and on a population of the squamous cells within the embryonic endoderm (EE) (from 7.3–8.0 dpc) [13], we next evaluated the impact of cubilin deficiency on these embryonic endodermal tissues. Examination of homozygous 8–8.5 dpc embryos revealed a number of endodermal anomalies (Fig. 7). In place of a normal squamous definitive endoderm (Fig. 7C), the mutant embryos had both cuboidal and columnar cells arranged in a stratified cuboidal epithelium (Fig. 7D, *arrow*) as well as simple columnar epithelium (not shown). Additionally, mutant cells in these epithelia had apical processes that may be either microvilli or cilia. Such luminal surface structures are normally not found on EE cells. Based on the findings, the absence of cubilin inhibits the formation of an epithelium morphologically comparable to that of a normal definitive endoderm at this stage, however, it remains to be determined whether this is a result of a defect in the specification and differentiation of the definitive endoderm.

**Figure 8:**
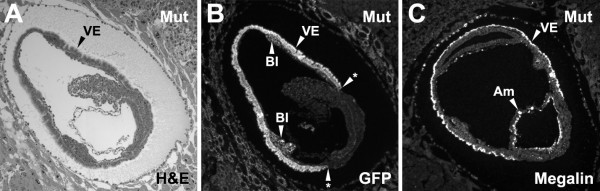
**Green fluorescent protein and megalin expression in yolk sac visceral endoderm of mutant embryos**. The cubilin gene targeting strategy (see Fig. 1) created a GFP reporter for *cubilin *locus expression. Shown in ***A ***is an H&E stained section of a homozygous 8.0 dpc embryo. Shown in ***B ***is anti-GFP fluorescence of a serial section to the one shown in A. Note that cells within the blood islands are expressing the GFP reporter. Shown in ***C ***is an anti-megalin stained 8.0 dpc mutant embryo. Embryos in panels A-B are oriented such that anterior is to the left and posterior is to the right. The embryo in C is oriented with anterior to the right and posterior to the left. VE, yolk sac visceral endoderm; BI, blood island; Am, amnion; Asterisk, points to the boundary of the VE and presumed EE.

Examination of the VE of homozygous 8–8.5 dpc embryos revealed it to also be morphologically abnormal. In contrast to the simple columnar VE of normal embryos, the VE of cubilin mutants was a stratified epithelium, comprised of cuboidal and columnar cells. Furthermore, mutant VE cells also lacked the large apical vacuoles that are characteristic of VE cells (Fig. 7E and 7F). Given the morphological similarities between the cells of mutant embryonic endoderm there was not a discernable demarcation between the normally distinct EE and VE epithelia. However, a clear demarcation between the VE and an epithelium that would normally be EE was evident upon immunohistological analysis of GFP reporter expression (from the EGFP cassette inserted into the cubilin gene) and megalin expression in mutant 8.0 dpc embryos (Fig. 8). This indicated that although being morphologically similar, the VE and presumed EE of mutants were distinct at the molecular level. Furthermore, the fact that megalin was appropriately expressed on the apical surfaces of the mutant VE indicated that cubilin is not required to direct cell surface trafficking of megalin.

### The visceral endoderm of homozygous embryos does not mediate uptake of maternal-derived HDL

**Figure 9:**
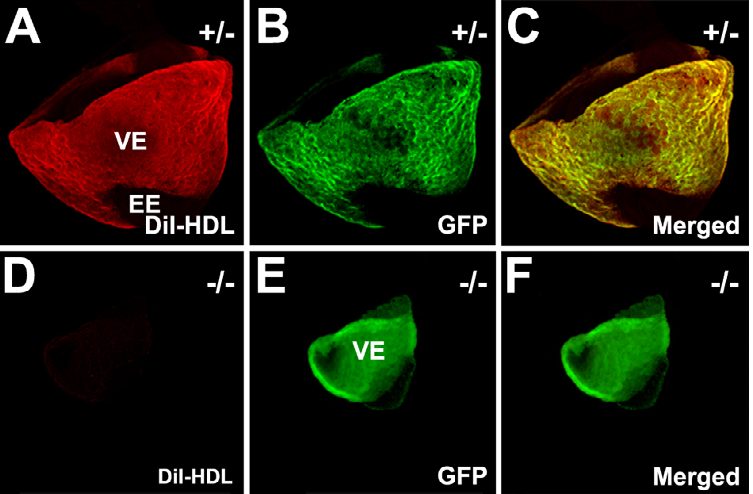
**Maternally derived HDL is not taken up by the yolk sac visceral endoderm of cubilin-deficient embryos**. Shown are confocal micrographs of a heterozygous 8.0 dpc embryo (***A-C***) and a homozygous littermate (***D-F***) isolated 1 hour after DiI-HDL was infused into the mother. The distal portion of the VE was removed from all embryos shown. VE, visceral endoderm; EE, embryonic endoderm.

A normal function of VE is to mediate uptake of maternal-derived HDL [8]. We therefore evaluated the capacity of cubilin-deficient VE to mediate uptake of maternal-derived HDL. As shown in Figure 9, the VE of heterozygous 8.0 dpc embryos from DiI-HDL-infused mothers showed strong DiI labeling within VE cells. DiI label in these embryos was present exclusively in VE which is the only yolk sac endodermal component expressing cubilin as evidenced by expression of the cubilin gene reporter, EGFP. By contrast, there was no detectable DiI labeling of the VE of homozygous embryos (Fig. 9D–F). These findings indicate that VE uptake of HDL is a cubilin dependent process.

## Discussion

Considering that cubilin forms a complex with AMN (forming the cubam complex), that AMN and cubilin are co-expressed in absorptive epithelial including the VE [11], and that AMN is required to mediate key aspects of cubilin function (e.g., membrane association, apical sorting) [11,12], it is not surprising that the phenotype of AMN-deficient mutants [18] closely resembles that of the cubilin nulls described. Like the cubilin null embryos, AMN-deficient embryos display arrested development, with most mutants not advancing morphologically beyond a normal 8–8.5 dpc embryo. On a mixed 129Sv/C57BL/6 genetic background, both mutants form an amnion. General mesoderm formation in the cubilin and AMN mutant embryos appears normal in that both mutants form an allantois, blood islands, yolk sac blood vessels and a heart. However, both mutants fail to form somites, indicating that somitogenesis is in some manner defective (e.g., insufficient paraxial mesoderm or defective condensation and/or segmentation). Both mutants also display endoderm defects as evidenced by the inability of cubilin-deficient VE to facilitate uptake of maternal HDL and the inability of AMN-deficient VE and EE to support wild-type epiblast development in chimeric embryos [18]. The morphological abnormalities of VE and definitive endoderm observed in the cubilin mutants also highlight the importance of cubilin in formation and/or maintenance of these epithelia. Such morphological abnormalities were not reported in the phenotype of AMN-deficient mutants. It remains to be established whether cubilin and AMN are playing roles in the specification and differentiation of the definitive endoderm, which forms during gastrulation by the recruitment of epiblast cells through the primitive streak [19].

The close similarities between the phenotypes of mouse *amn *and *cubilin *mutants also suggest that both genes act co-operatively in the VE to support embryonic growth and to control the formation of paraxial mesoderm. How AMN and cubilin mechanistically mediate these processes remains to be resolved. The endocytic function of the *cubam *complex and its apical expression in VE supports the possibility that it functions in the transport of vital maternal nutrients/factors. The spectrum of maternally derived nutrients/factors transported by VE *cubam *is potentially quite broad considering the multi-ligand binding capacity of cubilin which includes albumin, transferrin, immunoglobulin light chains, vitamin D-binding protein, myoglobin, galectin-3, Clara cell secretory protein, apoA-I and HDL [6]. Furthermore, at least one cubilin ligand, HDL, is a complex of multiple factors including apolipoproteins, phospholipids, cholesterol and lipid soluble vitamins such as retinol, which is converted to retinoic acid through the action of retinaldehyde dehydrogenase-2 (Raldh2). Indeed, genetic deficiency of mouse Raldh2 leads to early embryo lethality (~10.5 dpc), absence or reduction in somites, yolk sac vascular defects, enlarged heart and a failure of embryos to undergo axial rotation [20,21], all similar to *cubilin *and *amn *mutants.

By contrast to the developmental anomalies and early lethality of the cubilin and amnionless nulls, megalin-deficient mice die perinatally and display abnormal morphogenesis of the forebrain (e.g., holoprosencephaly), lung and kidney [22]. Thus, despite the evidence that megalin functions together with cubilin and that it is co-expressed with cubilin in the VE [13], any joint function that these two proteins may have in the early embryo is evidently not vital. Interestingly, mice deficient in disabled-2 (dab2), an adaptor of megalin, display several of the abnormalities observed in cubilin nulls including disorganization of the VE and loss of apical vesicles [23]. However, dab2^-/- ^mutants arrest earlier in development than do cubilin nulls and do not undergo gastrulation. Dab2 has also been shown to be required for cubilin endocytosis in VE [24]. The earlier lethality of dab2 null embryos may be an indication that numerous endocytic receptors expressed in the VE including megalin, cubilin and perhaps amnionless are dependent on dab2.

The importance of maternal derived lipoproteins as a source of cholesterol for normal development is well established [25]. Through the use of cubilin antagonists, the ability of cubilin to mediate VE uptake of holoparticle HDL, HDL-associated cholesterol and apoA-I has been demonstrated in mouse embryos cultured *in vitro *[8,14]. Our findings provide *in vivo *evidence that early (8.0 dpc) embryonic uptake of HDL from the maternal circulation is a cubilin-dependent process of the VE. Given the fact that the VE expresses apoA-I and the major LDL constituent, apoB [26], it is likely that cholesterol and other maternal HDL-derived constituents taken up by VE cells via cubilin are incorporated into new HDL and LDL particles and subsequently delivered to the embryo. In support of this, there is evidence that nascent lipoprotein particles have been localized to the rough endoplasmic reticulum and secretory vesicles of the 9.5 dpc mouse yolk sac and seen in the pericellular space underlying the endoderm [27].

Despite the fact that cubilin and AMN deficiency in mice leads to embryonic lethality, mutations in human and canine *AMN *that cause impaired apical targeting [28] and function (i.e., malabsorption of vitamin B_12_) of the *cubam *complex, nevertheless have no effect on embryonic development. One explanation for this apparent paradox may relate to the fact that there are species differences in yolk sac function in rodents, dogs and humans. Another explanation comes from the studies of Tanner et al. [29] who have shown that mutations affecting exons 1–4 of human *AMN *(OMIM 261100, megaloblastic anemia, MGA1) do not prevent the production variant transcripts and polypeptides resulting from alternative transcriptional start sites and alternative translation initiation sites. Indeed, the two *AMN *mutants that cause premature termination of translation (14ΔG and 208-2A→G) also express an alternative AMN polypeptide of 40 kDa and several minor species of 44, 42 and 38 kDa [29]. All of the alternative AMN polypeptides would contain the chordin-like module, transmembrane domain and cytoplasmic domain, but would lack various lengths of the amino terminal portion of the full-length protein. While these alternative polypeptides apparently are missing regions required for vitamin B_12 _uptake, they evidently confer enough normal function to sustain embryonic development. How these alternative polypeptides function, particularly with respect to cubilin, remains to be established. One possibility is that they retain the ability to mediate trafficking of cubilin to the apical surfaces of epithelial cells. However, mutations of the canine AMN gene that disrupt cubilin intracellular trafficking have no apparent effect on embryonic development [28]. It is not known whether the canine AMN mutants express alternative AMN polypeptides similar to those observed in humans. If so, it is possible that the polypeptides possess some activity that overcomes the need for apical expression of the *cubam *complex to mediate normal development.

## Conclusion

The present study reveals an indispensable role for cubilin in mouse embryogenesis particularly in the formation of endoderm and somites. The findings also highlight the importance of cubilin in the process of maternal-embryo transport of HDL by the VE.

## Methods

### Targeting vector design and generation of mutant mice

Degenerate deoxyoligonucleotide primers 5'-CTICACCARCCICGIATG-3' and 5'-CCRTTRRATYTCRCAYTC-3' were synthesized based on cDNA sequences conserved in the 5' region of both rat cubilin (GI: 24475743) and human cubilin (GI: 3929528). Preparation of template cDNA from adult mouse kidney total RNA and PCR amplification were done using methods described previously [8]. Cycling parameters for PCR amplification were: 94°C for 5 min and then 30 cycles of 94°C for 1 min, 46°C for 1 min and 72°C for 1.5 min. The expected ~820 bp product was sequenced and found to have 93% identity with a 5' portion of the rat cDNA sequence.

The mouse cubilin cDNA was used as a probe to screen a 129-strain mouse genomic library (Genome Systems, Inc.) and a BAC clone (24267) was isolated. DNA sequencing of ~20 kb of the BAC clone was performed to characterize the intron-exon organization of the 5' portion of the cubilin gene, including the region containing exons 1–10. A search of GenBank showed that the sequence of BAC clone 24267 was contained within the mouse chromosome 2 genomic contig GI:82796355.

The 3' arm of cubilin replacement-type targeting vector was constructed from a 5.4-kb *EcoRI *fragment containing exons 7 and 8 of the mouse *cubilin *gene. The 5' arm was constructed from a 4.3 kb ApaI-NaeI fragment containing *cubilin *exon 1 disrupted by insertion of a enhanced green fluorescent protein (EGFP)-N1 (Clontech, Mountain View, CA), *neo*^R ^(loxP floxed) cassette 33 nucleotides 5' to the first ATG (see Additional file 1). A *tk *negative selection cassette was placed at the 5' end of this construct. The resulting targeting vector was linearized by *NotI *digestion and electroporated into murine 129/Sv-strain embryonic stem (ES) cells. After electroporation of the construct and G418 positive selection and 1-(2-deoxy-2-fluoro-8-d-arabinofuranosyl)-5-iodouracil (FIAU) negative selection, clones having the desired homologous recombination were identified by Southern analysis (Fig. 1C). One of the targeted ES clones was injected into C57BL/6J blastocysts that were then transferred to foster mothers to obtain chimeric mice. Two male chimeras were obtained that were germ line competent. Southern analysis confirmed that offspring from these mice contained the properly targeted *cubilin *allele. Mice used in this study were maintained on a mixed 129Sv/C57BL/6 genetic background.

Genotypes of progeny from heterozygote intercrosses were determined by PCR. For embryos between 7.5 and 8.5 days postcoitum (dpc) DNA was isolated from the whole embryo and used in PCR. For embryos between 9.5 and 14.5 dpc, DNA for genotyping was isolated from the yolk sac. Two primer pairs were used for PCR-based genotyping. To detect the wild-type *cubilin *allele, PCR was performed with tail clip genomic DNA preparations using a *cubilin *sense strand primer, 5'-GCCAAGTAGACCAGGCTGAC-3' (residues 10422223–10422242 in GI: 82796355, and an antisense strand primer, 5'-GCTTCTGAGCCCAGTGAAAC-3' (residues 10422576–10422595 in GI: 82796355). Cycling parameters for PCR amplification were: 98°C for 5 min and then 40 cycles of 98°C for 0.5 min, 55°C for 1 min and 72°C for 1 min. The expected amplicon size is 373 bp. To detect the targeted *cubilin *allele, PCR reactions were performed with an EGFP sense strand primer, 5'-CCTGAAGTTCATCTGCACCA-3' (residues 810–829 in GI:1377911), and EGFP antisense strand primer 5'-TGCTCAGGTAGTGGTTGTCG-3' (residues 1288–1269 in GI:1377911). Cycling parameters for PCR amplification were: 98°C for 5 min and then 40 cycles of 98°C for 0.5 min, 55°C for 1 min and 72°C for 1 min. The expected amplicon size is 478 bp.

For embryos from intercross heterozygous matings that were paraffin embedded within maternal tissue, the assignment of 'mutant' was based on two criteria: 1) by the absence of immunologically detectable cubilin (anti-cubilin T-16 from Santa Cruz Biotechnology, Santa Cruz, CA) in sections of the paraffin embedded embryos; and 2) the distinct mutant morphological phenotype corresponding to that of embryos confirmed by genotypic analysis to be homozygous nulls.

### Immunohistochemistry and histology

Embryos were harvested from pregnant females following timed cubilin+/- intercross matings, and fixed for 40 min in 4% paraformaldehyde/PBS. Hematoxylin and eosin (H&E) staining of paraffin embedded embryo sections (7 μm) was performed using standard techniques. Whole-mount immunohistochemistry for the PECAM1/CD31 was performed as described previously [30]. Antibodies to PECAM (clone Mec-13.3) were purchased from BD PharMingen (San Diego, CA). For GFP and megalin immunohistological staining, sections were stained with rabbit antibodies to GFP purchased from Abcam (Cambridge, MA) or rabbit megalin cytoplasmic domain antibodies described previously [13]. Fluorescently conjugated secondary antibodies were purchased from Jackson ImmunoResearch Labs (West Grove, PA). Stained tissue sections were analyzed using a Leica DMR research-grade microscope equipped with Leica objectives and a SPOT-RT camera (Diagnostic Instruments, Sterling Heights, MI).

### RT-PCR analysis

Total RNA was isolated from 8.5 dpc embryos from heterozygous matings using Trizol (Invitrogen). Template cDNA was prepared from 1 mg RNA using Superscript II Reverse Transcriptase (Invitrogen). To assess cubilin transcript levels two sets of PCR reactions were performed using a primer pair targeting the 5' end of the transcript and one targeting the 3' end. The 5' primer pair consisted of the forward primer 5'-ATGATGATGACCTTGGCGAATG-3' (residues 275–296 (exon 2) in GI: 94365995) and the reverse primer 5'-GCAGCCAAAGGGTGTTCCAG-3' (residues 587–606 (exon 6) in GI: 94365995). Cycling parameters for PCR amplification were: 98°C for 5 min and then 40 cycles of 98°C for 0.5 min, 58°C for 1 min and 72°C for 1 min. The expected amplicon size is 332 bp. The 3' primer pair consisted of the forward primer 5'-TCTCATACACCAACTACCCC-3' (residues 9221–9240 (exon 58) in GI: 94365995) and the reverse primer 5'-AGCAGTCTTGTGAGGGCAGC-3' (residues 10041–10060 (exon 62) in GI: 94365995). Cycling parameters for PCR amplification were: 98°C for 5 min and then 40 cycles of 98°C for 0.5 min, 58°C for 1 min and 72°C for 1 min. The expected amplicon size is 840 bp. To assess GAPDH transcript levels PCR was performed using a forward primer 5'-CGGTGTGAACGGATTTGGC-3' (residues 70–88 in GI: 47607489) and the reverse primer 5'-GCAGTGATGGCATGGACTGT-3' (residues 581–600 in GI: 47607489). Cycling parameters for PCR amplification were: 98°C for 5 min and then 40 cycles of 98°C for 0.5 min, 54°C for 1 min and 72°C for 1 min. The expected amplicon size is 531 bp. To assess b-actin transcript levels a forward primer 5'-CGGGACCTGACAGACTACCTC-3' (residues 627–647 in GI: 6671508) and the reverse primer 5'-AACCGCTCGTTGCCAATA-3' (residues 827–844 in GI: 6671508) were used. Cycling parameters for PCR amplification were: 98°C for 5 min and then 40 cycles of 98°C for 0.5 min, 55°C for 1 min and 72°C for 1 min. The expected amplicon size is 218 bp. To assess EGFP transcript levels a forward primer 5'-ACGTAAACGGCCACAAGTTC-3' (residues 743–762 in GI: 1377911) and the reverse primer 5'-AAGTCGTGCTGCTTCATGTG-3' (residues 910–929 in GI: 1377911) were used. Cycling parameters for PCR amplification were: 98°C for 5 min and then 40 cycles of 98°C for 0.5 min, 58°C for 1 min and 72°C for 1 min. The expected amplicon size is 187 bp.

### Analysis of embryonic uptake of maternal derived HDL

Human DiI-labeled HDL (DiI-HDL) was purchased from Biomedical Technologies (Stoughton, MA). DiI-HDL was depleted of apoE-HDL and other heparin-binding particles according to Oram [31], dialyzed against 150 mM NaCl, 50 mM Tris pH 7.4 (TBS) containing 0.3 mM EDTA and filter-sterilized. Lipoprotein concentration was determined by BCA protein assay (Pierce, Rockford, IL). DiI-HDL was diluted into PBS to a final concentration of 0.265 mg protein/ml and 75–125 μl was infused into the saphenous vein of pregnant heterozygous mice at 8.0 dpc according to the procedure of Hem et al. [32]. One hour after infusion, embryos were isolated free of parietal endoderm and analyzed by confocal microscopy.

## Abbreviations

VE, visceral endoderm; EE, embryonic endoderm; Am, amnion; HDL, high density lipoprotein; DiI-HDL, DiI-labeled HDL; apoA-I, apolipoprotein A-I; AMN, amnionless; Cbl, cobalamin; dpc, days postcoitum; H&E, Hematoxylin and eosin; EGFP, enhanced green fluorescent protein.

## Authors' contributions

B. T. S. generated the targeting vector, carried out analyses of knockout mice, contributed to experimental design and helped draft the manuscript. J. C. M. carried out morphological and genotypic analyses of the null embryo phenotype, maternal HDL transport experiments and writing of the manuscript. P. A. F. assisted with embryo isolation, immunohistological analysis and embryo imaging. J. L. B. performed degenerate amplification of mouse cubilin 5' cDNA sequences, isolated and characterized the BAC used to generate the targeting vector and directed cubilin 5' RACE analysis. M. A. C. assisted with the design of the targeting vector. D. D. S. performed vector electroporation, selection of targeted ES cells, blastocyst injection and implantation. C. J. D. assisted with the phenotypic characterization of null embryos and writing of the manuscript. W. S. A. conceived of the study, contributed to experimental design and interpretation of results, and coordinated the project and writing of the manuscript.

## Supplementary Material

Additional File 1 Mapping of cubilin transcription initiation sites. The file contains findings from 5' RACE experiments used to position the EGFP reporter sequence within the 5' UTR of exon 1 of the reporter knock-in/KO targeting construct. Click here for file

## References

[B1] Christensen EI, Birn H (2002). Megalin and cubilin: multifunctional endocytic receptors. Nat Rev Mol Cell Biol.

[B2] Seetharam B, Christensen EI, Moestrup SK, Hammond TG, Verroust PJ (1997). Identification of rat yolk sac target protein of teratogenic antibodies, gp280, as intrinsic factor-cobalamin receptor. J Clin Invest.

[B3] Kozyraki R, Kristiansen M, Silahtaroglu A, Hansen C, Jacobsen C, Tommerup N, Verroust PJ, Moestrup SK (1998). The human intrinsic factor-vitamin B12 receptor, cubilin: molecular characterization and chromosomal mapping of the gene to 10p within the autosomal recessive megaloblastic anemia (MGA1) region.. Blood.

[B4] Hammad SM, Stefansson S, Twal WO, Drake CJ, Fleming P, Remaley A, Brewer HBJ, Argraves WS (1999). Cubilin, the endocytic receptor for intrinsic factor-vitamin B(12) complex, mediates high-density lipoprotein holoparticle endocytosis. Proc Natl Acad Sci U S A.

[B5] Kozyraki R, Fyfe J, Kristiansen M, Gerdes C, Jacobsen C, Cui S, Christensen EI, Aminoff M, de la Chapelle A, Krahe R, Verroust PJ, Moestrup SK (1999). The intrinsic factor-vitamin B12 receptor, cubilin, is a high-affinity apolipoprotein A-I receptor facilitating endocytosis of high-density lipoprotein. Nat Med.

[B6] Barth JL, Argraves WS (2001). Cubilin and megalin: partners in lipoprotein and vitamin metabolism. Trends Cardiovasc Med.

[B7] Moestrup SK, Kozyraki R, Kristiansen M, Kaysen JH, Rasmussen HH, Brault D, Pontillon F, Goda FO, Christensen EI, Hammond TG, Verroust PJ (1998). The intrinsic factor-vitamin B12 receptor and target of teratogenic antibodies is a megalin-binding peripheral membrane protein with homology to developmental proteins. J Biol Chem.

[B8] Hammad SM, Barth JL, Knaak C, Argraves WS (2000). Megalin acts in concert with cubilin to mediate endocytosis of high density lipoproteins. J Biol Chem.

[B9] Yammani RR, Sharma M, Seetharam S, Moulder JE, Dahms NM, Seetharam B (2002). Loss of albumin and megalin binding to renal cubilin in rats results in albuminuria after total body irradiation. Am J Physiol Regul Integr Comp Physiol.

[B10] Fyfe JC, Madsen M, Hojrup P, Christensen EI, Tanner SM, de la Chapelle A, He Q, Moestrup SK (2004). The functional cobalamin (vitamin B12)-intrinsic factor receptor is a novel complex of cubilin and amnionless. Blood.

[B11] Strope S, Rivi R, Metzger T, Manova K, Lacy E (2004). Mouse amnionless, which is required for primitive streak assembly, mediates cell-surface localization and endocytic function of cubilin on visceral endoderm and kidney proximal tubules. Development.

[B12] Coudroy G, Gburek J, Kozyraki R, Madsen M, Trugnan G, Moestrup SK, Verroust PJ, Maurice M (2005). Contribution of cubilin and amnionless to processing and membrane targeting of cubilin-amnionless complex. J Am Soc Nephrol.

[B13] Drake CJ, Fleming PA, Larue AC, Barth JL, Chintalapudi MR, Argraves WS (2004). Differential distribution of cubilin and megalin expression in the mouse embryo. Anat Rec.

[B14] Assemat E, Vinot S, Gofflot F, Linsel-Nitschke P, Illien F, Chatelet F, Verroust P, Louvet-Vallee S, Rinninger F, Kozyraki R (2005). Expression and role of cubilin in the internalization of nutrients during the peri-implantation development of the rodent embryo. Biol Reprod.

[B15] Sahali D, Mulliez N, Chatelet F, Dupuis R, Ronco P, Verroust P (1988). Characterization of a 280-kD protein restricted to the coated pits of the renal brush border and the epithelial cells of the yolk sac. Teratogenic effect of the specific monoclonal antibodies.. J Exp Med.

[B16] Sahali D, Mulliez N, Chatelet F, Laurent-Winter C, Citadelle D, Roux C, Ronco P, Verroust P (1992). Coexpression in humans by kidney and fetal envelopes of a 280 kDa-coated pit-restricted protein. Similarity with the murine target of teratogenic antibodies.. Am J Pathol.

[B17] Argraves WS, Larue AC, Fleming PA, Drake CJ (2002). VEGF signaling is required for the assembly but not the maintenance of embryonic blood vessels. Dev Dyn.

[B18] Tomihara-Newberger C, Haub O, Lee HG, Soares V, Manova K, Lacy E (1998). The amn gene product is required in extraembryonic tissues for the generation of middle primitive streak derivatives. Dev Biol.

[B19] Lewis SL, Tam PP (2006). Definitive endoderm of the mouse embryo: Formation, cell fates, and morphogenetic function. Dev Dyn.

[B20] Niederreither K, Subbarayan V, Dolle P, Chambon P (1999). Embryonic retinoic acid synthesis is essential for early mouse post-implantation development. Nat Genet.

[B21] Sirbu IO, Duester G (2006). Retinoic-acid signalling in node ectoderm and posterior neural plate directs left-right patterning of somitic mesoderm. Nat Cell Biol.

[B22] Willnow TE, Hilpert J, Armstrong SA, Rohlmann A, Hammer RE, Burns DK, Herz J (1996). Defective forebrain development in mice lacking gp330/megalin. Proc Natl Acad Sci U S A.

[B23] Yang DH, Smith ER, Roland IH, Sheng Z, He J, Martin WD, Hamilton TC, Lambeth JD, Xu XX (2002). Disabled-2 is essential for endodermal cell positioning and structure formation during mouse embryogenesis. Dev Biol.

[B24] Maurer ME, Cooper JA (2005). Endocytosis of megalin by visceral endoderm cells requires the Dab2 adaptor protein. J Cell Sci.

[B25] Woollett LA (2005). Maternal cholesterol in fetal development: transport of cholesterol from the maternal to the fetal circulation. Am J Clin Nutr.

[B26] Shi WK, Heath JK (1984). Apolipoprotein expression by murine visceral yolk sac endoderm. J Embryol Exp Morphol.

[B27] Farese RVJ, Cases S, Ruland SL, Kayden HJ, Wong JS, Young SG, Hamilton RL (1996). A novel function for apolipoprotein B: lipoprotein synthesis in the yolk sac is critical for maternal-fetal lipid transport in mice. J Lipid Res.

[B28] He Q, Madsen M, Kilkenney A, Gregory B, Christensen EI, Vorum H, Hojrup P, Schaffer AA, Kirkness EF, Tanner SM, de la Chapelle A, Giger U, Moestrup SK, Fyfe JC (2005). Amnionless function is required for cubilin brush-border expression and intrinsic factor-cobalamin (vitamin B12) absorption in vivo. Blood.

[B29] Tanner SM, Aminoff M, Wright FA, Liyanarachchi S, Kuronen M, Saarinen A, Massika O, Mandel H, Broch H, de la Chapelle A (2003). Amnionless, essential for mouse gastrulation, is mutated in recessive hereditary megaloblastic anemia. Nat Genet.

[B30] Drake CJ, Fleming PA (2000). Vasculogenesis in the day 6.5 to 9.5 mouse embryo. Blood.

[B31] Oram JF (1986). Receptor-mediated transport of cholesterol between cultured cells and high-density lipoproteins. Methods Enzymol.

[B32] Hem A, Smith AJ, Solberg P (1998). Saphenous vein puncture for blood sampling of the mouse, rat, hamster, gerbil, guinea pig, ferret and mink. Lab Anim.

[B33] Kristiansen M, Kozyraki R, Jacobsen C, Nexo E, Verroust PJ, Moestrup SK (1999). Molecular dissection of the intrinsic factor-vitamin B12 receptor, cubilin, discloses regions important for membrane association and ligand binding. J Biol Chem.

[B34] Yammani RR, Seetharam S, Seetharam B (2001). Identification and characterization of two distinct ligand binding regions of cubilin. J Biol Chem.

