# Supplementary material for: Targeted disruption of cubilin reveals essential developmental roles in the structure and function of endoderm and in somite formation
Source: BMC Dev Biol. 2006 Jun 20;6:30. doi: 10.1186/1471-213X-6-30 (PMC1533814; doi:10.1186/1471-213X-6-30)
Supplement: Additional File 1 — Mapping of cubilin transcription initiation sites. The file contains findings from 5' RACE experiments used to position the EGFP reporter sequence within the 5' UTR of exon 1 of the reporter knock-in/KO targeting construct. [file 1471-213X-6-30-S1.doc]

*Additional file 1*: 5’ RACE was performed to map the cubilin transcription initiation site using RNA isolated from adult mouse ileum and kidney and from embryonic yolk sac. The results showed that the position of transcriptional initiation varies depending on the tissue type. In RNA from kidney the start site was 49 bp upstream from the ATG. In RNA from ileum the start site was variable, occurring at either 193, 163 or 135 bp upstream from the ATG. In RNA from yolk sac the start site also varied occurring at either 101 or 69 bp upstream from the ATG. This information was used to help decide where to position the EGFP reporter sequence within the 5’ UTR of exon 1 of our reporter knock-in/KO targeting construct. The cassette was placed at position 10431004 (GI: 82796355), 33 residues upstream of the ATG). This location was 16 residues downstream from the most 3’ transcription start site (residue 10431020 in GI: 82796355). This was done to assure that EGFP expression would occur in the various sites of endogenous cubilin expression.
